# Analysing chemical attraction of gravid *Anopheles gambiae sensu stricto* with modified BG-Sentinel traps

**DOI:** 10.1186/s13071-015-0916-0

**Published:** 2015-06-03

**Authors:** Michael N. Okal, Manuela Herrera-Varela, Paul Ouma, Baldwyn Torto, Steven W. Lindsay, Jenny M. Lindh, Ulrike Fillinger

**Affiliations:** Disease Control Department, London School of Hygiene and Tropical Medicine, Keppel Street, London, WC1E 7HT UK; International Centre of Insect Physiology and Ecology, P.O. Box 30772–00100, Nairobi, Kenya; School of Biological and Biomedical Sciences, Science Laboratories, Durham University, Durham, DH1 3LE UK; Royal Institute of Technology, Stockholm, S-100 44 Sweden

**Keywords:** *Anopheles gambiae*, Oviposition, Breeding site, Choice-tests, BG-Sentinel mosquito trap, Attractants, Semiochemicals

## Abstract

**Background:**

Cues that guide gravid *Anopheles gambiae sensu lato* to oviposition sites can be manipulated to create new strategies for monitoring and controlling malaria vectors. However, progress towards identifying such cues is slow in part due to the lack of appropriate tools for investigating long-range attraction to putative oviposition substrates. This study aimed to develop a relatively easy-to-use bioassay system that can effectively analyse chemical attraction of gravid *Anopheles gambiae* sensu stricto.

**Methods:**

BG-Sentinel™ mosquito traps that use fans to dispense odourants were modified to contain aqueous substrates. Choice tests with two identical traps set in an 80 m^2^ screened semi-field system were used to analyse the catch efficacy of the traps and the effectiveness of the bioassay. A different batch of 200 gravid *An. gambiae s.s.* was released on every experimental night. Choices tested were (1) distilled versus distilled water (baseline) and (2) distilled water versus soil infusion. Further, comparisons were made of distilled water and soil infusions both containing 150 g/l of Sodium Chloride (NaCl). Sodium Chloride is known to affect the release rate of volatiles from organic substrates.

**Results:**

When both traps contained distilled water, 45 % (95 confidence interval (CI) 33–57 %) of all released mosquitoes were trapped. The proportion increased to 84 % (95 CI 73–91 %) when traps contained soil infusions. In choice tests, a gravid female was twice as likely to be trapped in the test trap with soil infusion as in the trap with distilled water (odds ratio (OR) 1.8, 95 % CI 1.3–2.6). Furthermore, the attraction of gravid females towards the test trap with infusion more than tripled (OR 3.4, 95 % CI 2.4–4.8) when salt was added to the substrates.

**Conclusion:**

Minor modifications of the BG-Sentinel™ mosquito trap turned it into a powerful bioassay tool for evaluating the orientation of gravid mosquitoes to putative oviposition substrates using olfaction. This study describes a useful tool for investigating olfactory attraction of gravid *An. gambiae s.s.* and provides additional evidence that gravid mosquitoes of this species are attracted to and can be baited with attractive substrates such as organic infusions over a distance of several metres.

## Background

Malaria still causes considerable human morbidity and mortality in spite of concerted control efforts that have resulted in its steady decline in the last decade [1]. Effective interventions need to be scaled up [2] and new approaches added to the armamentarium for controlling the disease and its vectors [3, 4]. The two front-line interventions for controlling malaria vectors in Africa, long-lasting insecticidal nets (LLINs) and indoor residual spraying (IRS), have exploited the indoor resting and host-seeking behaviour and the susceptibility of vectors to insecticides. These tools led to a major reduction of 29 % in malaria cases worldwide [2] justifying efforts to scale up LLINs and IRS. However, because of the growing problem of insecticide resistance [5–7], increasing importance of outdoor-biting vector populations [4] as well as heritable and plastic changes in vector behaviour in response to control [4, 8–12] the effectiveness of existing approaches may be compromised and there is a growing need for additional strategies against malaria vectors.

In spite of many anticipated challenges and limitations [13], mass trapping of mosquitoes using synthetic attractant baits offers an exciting possibility for an eco-friendly, sustainable complementary strategy for monitoring and controlling disease vectors. Strategies that target gravid mosquitoes would be useful for vectors that rest and bite both indoors and outdoors irrespective of their state of insecticide resistance. Extensive behavioural and chemical ecology studies on host-seeking members of the *Anopheles gambiae* species complex (including *Anopheles gambiae* sensu stricto *(s.s.)* and *Anopheles arabiensis*) which are the primary vectors of malaria in sub-Saharan Africa, have led to considerable progress towards identification of odourants from skin emanations of humans and other primary blood meal hosts [14, 15] and host plants [16]. These volatiles have been incorporated into baits and tested in traps [16, 17]. In contrast, very little is known about the cues that gravid females of these species use to find and orientate towards an aquatic habitat to lay their eggs. Whilst a range of physical and chemical cues associated with the aquatic habitat have been suggested [18–22] empirical evidence is scarce and restricted to cage and electrophysiological studies not least due to the lack of appropriate bioassay tools.

Malaria vectors bite human hosts for vertebrate blood that they require for ovarian development. The malaria parasite (*Plasmodium sp*.) inadvertently imbibed with a blood meal will require at least eight days to complete the sexual stage within the mosquito [23, 24]. In theory, this period is punctuated by two or more oviposition cycles; a period when gravid mosquitoes look for suitable breeding sites, lay eggs and recommence the search for new hosts to bite for blood [25]. Targeting gravid vectors while they forage for aquatic habitats for their offspring would thus conceivably provide an effective approach to prevent the ultimate infective bites of parous mosquitoes and reduce overall vector population densities. Relevant oviposition cues that malaria vectors use to detect, find and evaluate potential breeding sites could be identified and exploited in various attract and kill strategies by luring female mosquitoes either into traps or into contact with insecticides [26].

Laboratory evidence shows that gravid females of the *An. gambiae* complex discriminate between different oviposition substrates. They are able to detect substrates with different levels of moisture and relative humidity [27, 28] and the presence or absence of bacteria [18, 29, 30]. A recent study demonstrated that at short-range gravid *An. gambiae s.s.* can avoid or select substrates using olfactory cues [31]. In another comparable laboratory study one synthetic odourant, two-propylphenol was shown to increase the egg-laying rate of *An. gambiae s.s.* in cage tests [32]. However, to the best of our knowledge no study has provided evidence that gravid females of the *An. gambiae* complex orient towards a suitable aquatic habitat over a distance of several metres using attractant chemical cues except using the bioassay here described.

The aim of the present study was to develop a simple bioassay for measuring olfactory orientation of gravid *An. gambiae s.s.* in the semi-field and to evaluate the response of gravid mosquitoes to soil infusions previously described [31] to increase the egg-laying rate of these species in small experimental cages. Laboratory studies have shown that the addition of inorganic salts to aqueous solutions can lead to a higher release of volatile organic compounds into the headspace of the solution, an effect that is known as salting-out [33–39]. For instance, Mozuraitis et al. [40] showed that the amount of volatiles detected in the headspace from oestrous urine of mares increased eight times when the urine sample was saturated with salt compared to samples without salt. This study used salt to alter the volatile profile of the infusions and measure the sensitivity of the newly developed tool to changes in the chemical headspaces of these infusions. As a result, an effective tool that has since been used to describe the first oviposition semiochemical for the species [41] was described in detail.

## Methods

### Study site

The study was carried out between March 2013 and February 2014 (time of sunset between 18.30 h and 19.00 h) at the International Centre of Insect Physiology and Ecology, Thomas Odhiambo Campus (icipe – TOC) at Mbita on the shores of Lake Victoria in western Kenya (0° 26’ 06.19” S, 34° 12’ 5313” E; altitude 1137 m above sea label). This area is characterised by a tropical climate with temperatures ranging between a mean minimum of 16 °C and a mean maximum of 28 °C and two rainy seasons each year between March and June and October and December.

### Mosquito preparation

The Mbita strain of *An. gambiae s.s*. reared at the icipe -TOC mosquito insectaries was used for all experiments. Temperature and relative humidity in the insectary varied between 25 and 28 °C and 68–75 %. About 300 female mosquitoes held in a 30 × 30 × 30 cm netting cage with an equal number of males of a similar age were provided with two blood-meals on two consecutive nights from a human arm. Mosquitoes were starved for 6 h before the blood-meal, which was offered for 15 min at 19:00 h. Mosquitoes that remained unfed after the first blood meal were removed from the cage. A piece of cotton (50 × 25 cm) saturated with distilled water and positioned on top of the cage ensured that mosquitoes remained hydrated throughout oogenesis. Mosquitoes were left unattended for two days after the second blood meal except for changing the 6 % glucose solution provided as energy source and saturating the cotton on the cage with water twice a day. Gravid mosquitoes were selected through visual inspection on the third day. Females were presumed gravid when they had an opaque and pale distended abdomen.

### Two-choice experiments

**Fig. 1:**
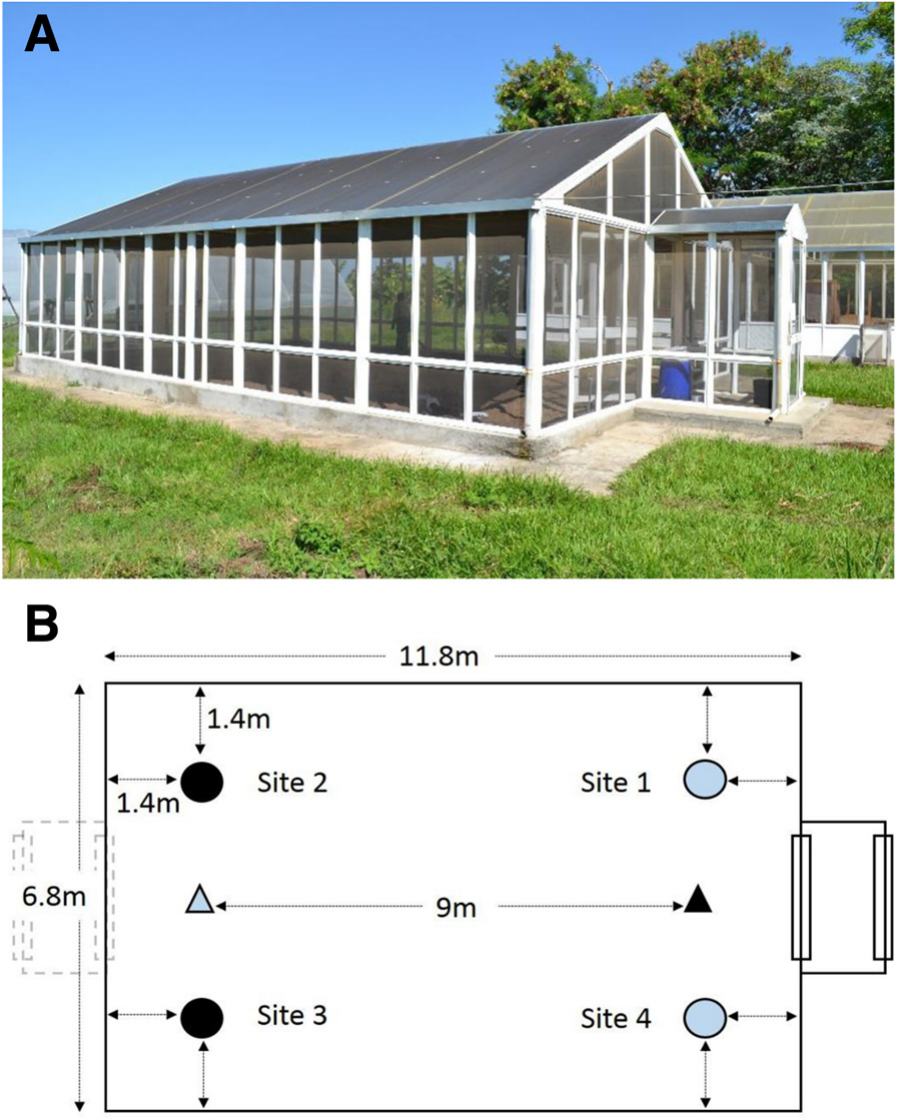
Semi-field system (**a**) and schematic diagram of trap positions and release sites (**b**) Trap positions are shown in circles and mosquito release points in triangles. Colour codes show corresponding trap positions and mosquito release points

Two-choice experiments were implemented under semi-field conditions (i.e., ambient temperature, humidity, light conditions) in a large netting-screened structure (black fibreglass gauze 1.7 × 1.5 mm); 6.8 m wide and 10.8 m long (semi-field system; Fig. 1). A netting ceiling was stretched across the cage 2.4 m above the ground (176.3 m^3^). The floor was covered with sand to a depth of 50 cm. A roof made from transparent polycarbonate sheets shielded the structure from rainfall. The rectangular floor plan (two long walls, two short walls) of the semi-field system provided for four possible trap positions. Each position was arbitrarily set in each corner 1.4 m from the nearest adjoining walls (Fig. 1). The two trap positions along the shorter walls of the semi-field system received approximately the same proportion of mosquitoes whilst there was large variability in catches when traps were set in diagonally opposite corners in preliminary tests (data not shown). To reduce the statistical noise in the system the two traps constituting the dual-choice were always both placed at one of the two randomly selected short walls of the semi-field system, (site 2 + 3 and site 1 + 4; Fig. 1). Gravid mosquitoes were released as far as possible from the traps near the opposite wall of the greenhouse, 9 m away from the two traps. The location of the traps and the position of the treatments were randomly assigned for every night of an experiment. 200 gravid *An. gambiae s.s*. mosquitoes were released into the semi-field system at 17:30 h. Previous cage experiments [28] showed that the local mosquito strain has its peak oviposition time early in the evening before 21:30 h. To assess the proportion of gravid females that respond within this period the trapping chambers of the traps were changed at 21:30 h and the second pair retrieved at 08:00 h. This allowed tallying of the number of mosquitoes that were trapped with each treatment before 21:30 h and between 21:30–08:00 h. Each experiment was carried out on 12 nights based on previous sample size considerations [42], so that trap A and B were in each possible location three times. With this sample size an increment of 20 % in the trap rate could be detected with 80 % power at the 5 % significance level.

### Modification of the Biogents (BG)-Sentinel™ mosquito trap into a gravid mosquito trap

**Fig. 2:**
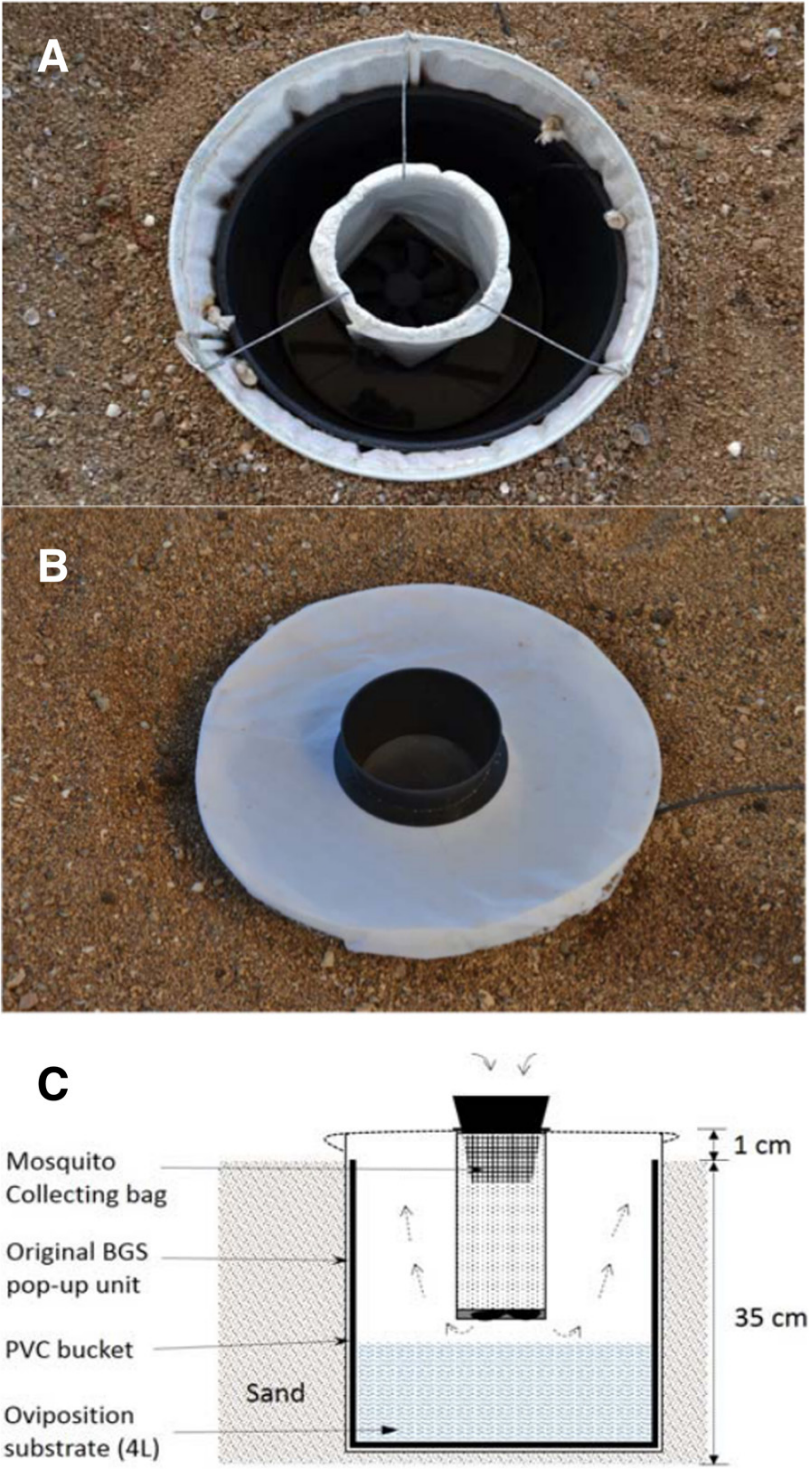
Modification and set-up of BG-Sentinel trap. (**a**) Interior showing bucket for holding aqueous solutions, (**b**) Complete trap (**c**) Cross-section of modified Biogents (BG)-Sentinel gravid mosquito trap

Commercially available BG-Sentinel™ mosquito traps (Biogents, Regensburg, Germany) were modified and tested in this study. This is an odour-baited trap that was originally designed for mass trapping of host-seeking virus vectors like *Aedes aegypti* and *Aedes albopictus* using a chemical lure based on human body emanations [43, 44]. One of the advantages of the trap is its size ‘which is large enough to incorporate additional attractants such as fragrant substances, small living animals, worn clothing, animal hairs, light and heat sources’ [45]. The trap consists of a collapsible, white fabric container with white gauze covering its opening. The trap is 36 cm in diameter and 40 cm high. In the middle of the gauze cover air is sucked into the trap through a black catch pipe by an electrical fan placed at its end. This draws approaching mosquitoes into a catch bag. Consequently, the air exits the trap through the gauze, generating ascending currents. The aim here was to include attractive oviposition media in the trap and to evaluate its catching efficiency under semi-field conditions. All oviposition sites of *Anopheles* mosquitoes are aquatic (or at least water saturated) and recent wind tunnel experiments suggested that water vapour is an important oviposition attractant for gravid *An. gambiae s.s*. [28]. Consequently, the BG-Sentinel was modified to hold 4 L of aqueous test substrates by inserting a tightly-fitting black plastic bucket (Pride, Mombasa, Kenya) 34 cm high and 30 cm inner diameter into the white fabric container. Since *An. gambiae s.s.* mosquitoes rarely oviposit in container-type habitats, the entire trap was dug into the ground leaving only 1 cm of it above ground (Fig. 2).

### Assessing the catching efficacy of the modified BG-Sentinel gravid mosquito trap

To evaluate if and how effectively gravid *An. gambiae s.s*. are attracted to oviposition substrates contained in the traps and to generate a baseline for comparison, an experiment was done where mosquitoes were presented with two traps with identical substrates. Both traps (trap A and B) were filled with 4 L of distilled water (Buyimpex Agencies LTD, Kenya), with the position of the traps allocated randomly. Fresh distilled water was used in every round of experiments.

### Analysing the response of gravid *Anopheles gambiae s.s.* to 6-day old soil infusions

Recently, a positive oviposition response of gravid *An. gambiae s.s*. to a 6-day old soil infusion made from water mixed with soil taken from a natural breeding site located at icipe-TOC was demonstrated in cage egg-count experiments and chemical cues suggested as the reason for this response [31]. However, egg-count experiments do not provide information on the nature of these chemical cues, which could either be volatile and attract mosquitoes from a distance or could be less volatile and act as contact stimulants [29, 46]. Here, the same soil was used to prepare infusions in the same way as before [31] and tested with the BG-Sentinel gravid mosquito trap. The silty clay loam top soil was dug from the same location as described by Herrera-Varela et al. [31] within the icipe*-*TOC compound and sun-dried for 24 h. 3 litres of dry soil were thoroughly mixed with 15 L of distilled water in a 20 L plastic tub and left undisturbed at ambient conditions, but protected from rain for 6 days except for daily water top-up to compensate for loss through evaporation. Throughout the six days the tub was covered with mosquito netting. Just before the experiments the infusion was filtered through a cotton cloth to remove large soil particles and small debris. Exactly 4 litres of the soil infusion were compared to an equal volume of distilled water in choice experiments in the semi-field system. Fresh batches of infusions and distilled water were used for every experimental night.

### Analysing the response of gravid mosquitoes to alterations in the headspace of 6-day old soil infusions

In order to be ideal for investigating attraction and formulating oviposition baits the bioassays used should be sensitive to changes in the release rate of odourants. An attempt was made to manipulate the release of odourants from soil infusion by adding NaCl and consequently investigating the sensitivity of the bioassay to small changes in the chemical headspace of the soil infusion. Following published data on salt concentrations [40], preliminary experiments were implemented where 45 g of NaCl was added to 300 ml of soil infusion (150 g/L) in a glass beaker and stirred to dissolve at room temperature. At this concentration small amounts of undissolved salt were observed settling at the bottom of the beaker. Hence, for choice experiments, 150 g of NaCl was added per litre of the test substrates (600 g/4 L) and stirred to dissolve 10–20 min before the onset of experiments at 17:30 h. Two experiments were implemented. First, choice tests were carried out with distilled water versus soil infusion, both with NaCl. Second, the attractiveness of soil infusion without NaCl was tested against soil infusion with NaCl.

### Data analysis

Data were analysed with generalised linear models with a binomial distribution and logit link function fitted to compare the probability of gravid *An. gambiae s.s*. being (1) collected in the test trap (trap B) compared with the total caught in both traps (trap A + trap B) to show substrate preference; (2) collected in both traps out of the total mosquitoes released (response rate); and (3) collected in both traps before 21.30 h out of the mosquitoes collected during the night (early responders). The underlying hypothesis of a choice bioassay is that when two equal choices are presented the response towards these choices is similar with odds of success of 1:1 (baseline or control). We expect that if an oviposition cue is presented that is either preferred or avoided by gravid females we will see a statistically significant diversion from the baseline. Consequently, the assay with two equal treatments served as reference. Initially, the trap location and the pair (wall) were included as fixed factors in the model to test for main effects and interactions. Since there were no significant associations with the outcome, these variables were excluded from the final models. The mean proportions of mosquitoes trapped in each treatment and their corresponding 95 % confidence intervals (CI) were calculated as the exponential of the parameter estimates for models with no intercept included. Data analyses were done using R statistical software version 3.00 with various functions contributed from the packages MASS, effects, epicalc, multcomp, lme4, gee, aod [47].

### Ethics statement

Ethical approval for this study was obtained from the Kenya Medical Research Institute’s Ethical Review Committee (Protocol no. 422).

## Results

### The modified BG-Sentinel gravid mosquito trap is an effective tool for analysing oviposition attraction of malaria vectors under semi-field conditions

**Fig. 3:**
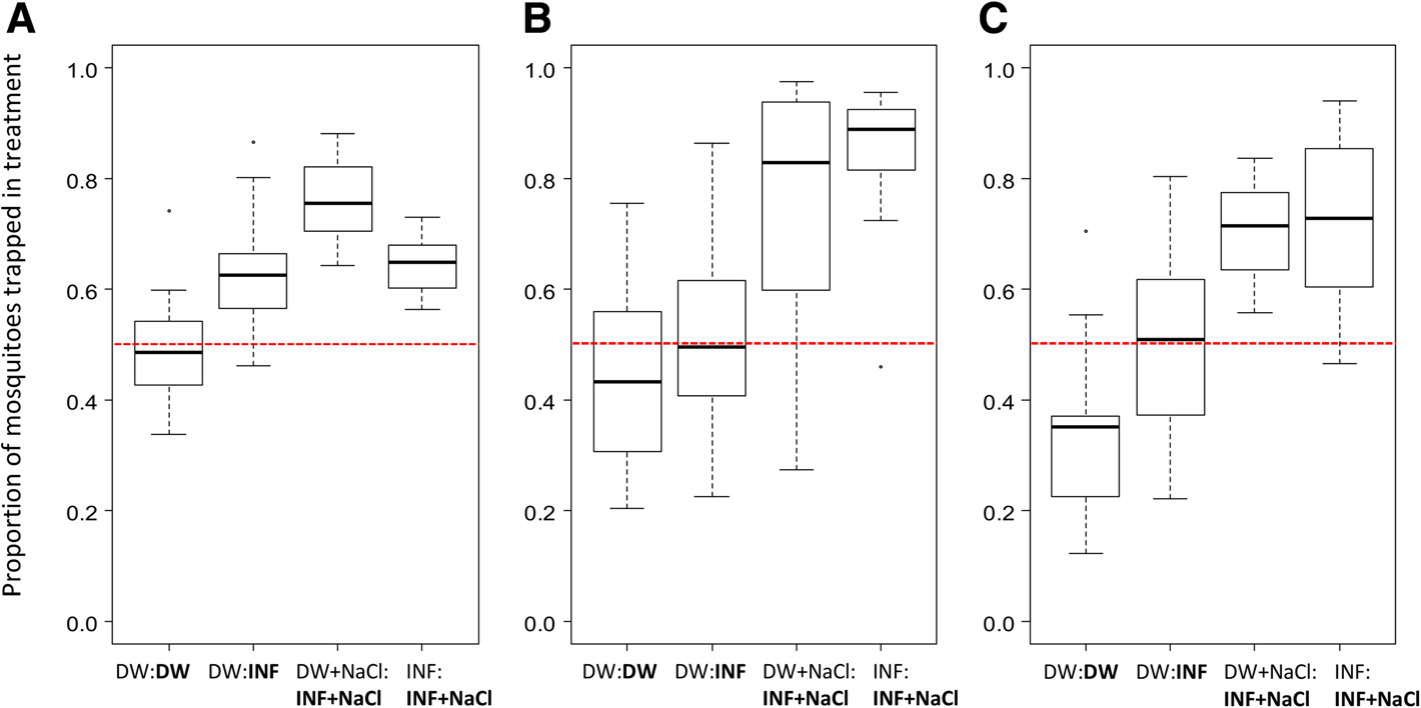
Explanatory data analyses of oviposition response of *Anopheles gambiae* sensu stricto to test substrates. Box-and-whisker plots indicating the median value by the central horizontal line and the lower and upper quartiles by corresponding ends of the box. The whiskers show the range of the data. Dots show outlying values. **a**. Proportion of females responding to the test substrate (INF = soil infusion, INFsalt = soil infusion with NaCl) compared to distilled water or infusion controls (DW = distilled water, DWsalt = distilled water with NaCl) in choice tests; **b**. Response rate of the females released (*N* = 200); **c**. Response of mosquitoes before 21:30 h out of the females trapped per night. The red line indicates 0.5 distribution

When two traps baited with distilled water were provided in choice tests, 45 % of the released mosquitoes were recovered. Importantly, trap A and B caught equal proportions of the mosquitoes (50, 95 CI 0.43–0.57 %), which validates the experimental design. Only about one third of all mosquitoes (36 95 CI 28–45 %) were trapped before 21:30 h (Fig. 3, Table 1).

**Table 1:** Oviposition response of gravid *Anopheles gambiae* sensu stricto to substrates in two-choice tests. Generalized linear model outputs

Oviposition substrates		Mean proportion	Odds ratio	*p-value*
Control (trap A)	Test (trap B)	(95 % CI)	(95 % CI)	
Proportion of gravid females trapped in test (trap B) in two choice experiments of the females trapped				
Distilled water	Distilled water	0.50 (0.43–0.57)	1	
Distilled water	Infusion	0.64 (0.58–0.70)	1.8 (1.3–2.6)	0.004
Distilled water + NaCl	Infusion + NaCl	0.77 (0.72–0.81)	3.4 (2.4–4.8)	<0.001
Infusion	Infusion + NaCl	0.67 (0.60–0.69)	1.8 (1.3–2.5)	0.001
Response rate of released gravid females during experiments with different substrate combinations				
Distilled-Distilled		0.45 (0.33–0.57)	1	
Distilled-Infusion		0.51 (0.39–0.63)	1.3 (0.6–2.5)	0.438
Distilled + NaCl – Infusion + NaCl		0.74 (0.63–0.84)	3.7 (1.8–7.5)	<0.001
Infusion - Infusion + NaCl		0.84 (0.73–0.91)	6.8 (3.1–15.0)	<0.001
Response of gravid females before 21:30 h of the females trapped during the night (early responders)				
Distilled-Distilled		0.36 (0.28–0.45)	1	
Distilled-Infusion		0.56 (0.46–0.67)	1.59 (1.11–2.29)	0.003
Distilled + NaCl – Infusion + NaCl		0.66 (0.58–0.74)	1.92 (1.38–2.68)	<0.001
Infusion - Infusion + NaCl		0.64 (0.54–0.73)	1.76 (1.29–2.44)	0.001

### Soil infusions contain odourants that attract gravid *Anopheles gambiae s.s.*

Gravid mosquitoes were twice as likely to be trapped in BG-sentinel gravid mosquito traps when the test trap (trap B) contained soil infusion as when the test trap contained distilled water in the bioassays with two equal choices (OR 1.8, 95 % CI 1.3–2.6). Moreover, adding NaCl increased the attractiveness of the infusion; females were 3.4 (95 % CI 2.4–4.8) times more likely to choose the infusion than distilled water. In direct comparisons of soil infusion with NaCl to soil infusions without NaCl, gravid females were nearly two times (OR 1.8, 95 CI 1.3–2.5 %) more likely to be collected in the trap containing the infusion with salt (Fig. 3, Table 1).

### The presence of attractive odourants in the semi-field system increases the response rate of gravid *Anopheles gambiae s.s.*

When salt-saturated infusions were present in one of the traps it was 3.7–6.8 times more likely for a mosquito to respond and be collected in either trap than when only distilled water was presented in both traps (Fig. 3, Table 1).

### Odourant cues from soil infusions prompt early oviposition site seeking in *Anopheles gambiae s.s.*

The presence of soil-infusion odourants doubled (OR 1.92, 95 % CI 1.38–2.68) the proportion of mosquitoes that responded before 21.30 h (Fig. 3, Table 1).

## Discussion

Minor modifications of the commercially available BG-Sentinel mosquito trap turned this trap into a valuable bioassay tool for evaluating the orientation of gravid mosquitoes to putative oviposition substrates using olfaction. The modified traps excluded any possible contact stimuli or visual cues (e.g., light reflections from water) from the test substrates and showed a strong discrimination effect enabling the detection of small differences (≥20 %) in the proportion of gravid mosquitoes attracted to one of two competing substrates (odourant blends). The BG-Sentinel mosquito trap is simple to set up and allows for rapid replacement of collection bags making it possible to evaluate the response of gravid mosquitoes at different periods during the night.

With this system we provide evidence that gravid females of the major malaria vector *An. gambiae s.s.* can use attractive odourant cues over at least nine metres to locate and choose between potential oviposition sites. Many studies have suggested the involvement of chemical cues in the selection of breeding sites [18, 19, 29, 31]. However, all of these studies were egg-count bioassays done in small cages (30 × 30 × 30 cm) with gravid mosquitoes released directly over test substrates. Consequently, none of the studies were able to prove attraction or describe an attractant, defined as cues that draw insects towards substrates [48, 49], or discount stimulation. This study shows that odourants from the soil infusions reported by Herrera-Varela et al. [31] attract gravid *An. gambiae s.s.*. Furthermore, our results show that oviposition attraction to odourant chemicals is affected by the strengths of the cue, as shown from the salting-out experiments. This observation is important if one wanted to use odour-baited traps for the surveillance or control of gravid mosquitoes since it indicates that olfactory cues can be manipulated to attract and mass trap gravid malaria vectors.

This study confirms earlier laboratory findings that gravid *An. gambiae s.s.* use water vapour to locate breeding sites [28]. Previous studies were done in small, closed laboratory systems, free of external odourants with standardized water vapour gradients [27, 28]. With the bioassay, where both traps contained distilled water only, this study provides evidence that malaria vectors use water vapour to orientate to substrates in more natural and fairly complex chemical spaces over larger distances. It is likely that water vapour is a general selective cue, but provides no information about the quality of the habitat, which might be the reason for the observed slow and low response of gravid females. In the complex chemical space of natural ecosystems it is unlikely that a species with such a highly developed olfactory apparatus should evolve to employ water vapour as the major cue for selecting favourable water bodies. Water vapour most likely indicates the presence of water bodies while chemical odourants enable mosquitoes to assess the suitability of this potential niche.

Based on the findings of this study it was hypothesised that the soil infusions tested contained at least one odourant that prompted habitat searching in gravid *An. gambiae s.s..* The odourant bouquet of soil infusions evidently compelled passive gravid *An. gambiae s.s.* mosquitoes to fly towards the potential oviposition sites, especially when the infusions were saturated with salt. This was in contrast to the response when only distilled water was provided. A similar response has been shown for host-seeking mosquitoes when exposed to carbon dioxide which triggers long-range directed host seeking flight in otherwise inactive females of the *An. gambiae* complex [50]*.* In nature such an odourant or collection of odourants would shorten the period for foraging for suitable aquatic sites by gravid mosquitoes. Gravid mosquitoes would use less energy and reduce the risk for mortality that is likely associated with prolonged habitat searching and altogether improve the odds for successful breeding.

This is the first study to exploit the principle of salting-out volatile chemicals to demonstrate the potential use of NaCl in behavioural bioassays to manipulate the odour profile of organic infusions. This study showed that adding NaCl to soil infusions increased the attraction of gravid *An. gambiae s.s.* to soil infusions two-fold and the response rate three-fold. This adds proof that *An. gambiae s.s.* respond to chemical cues in soil infusion. Whilst it cannot be excluded that the addition of salt affected the microbial organisms in the soil infusion and therefore changed the chemical composition of the volatile headspace, the increase in attractiveness of the already highly attractive soil infusion suggests that it is more likely that the addition of salt led to an increased release of already present attractive odours. Numerous studies using a wide range of inorganic salts have shown that these increase the concentration of volatile organic compounds (VOC) in the headspace above the salt containing solution [33–40]. The presence of salt decreases the solubility of the VOCs, which are pushed into the headspace. This effect is commonly known as the salting-out effect and can be quantified by the Setschenow constant [37, 51], which most frequently is positive (salting-out) but can also be negative (salting-in) [52, 53]. In preliminary studies (Lindh JM, personal communication) aimed at optimizing the collection of volatiles in the headspace above water from mosquito breeding sites, which should be similar in chemical composition to the soil infusion studied here, addition of NaCl increased the amount of the majority of the compounds detected and pushed many previously undetected organic compounds above the detection limit. This theoretically represents an inexpensive advancement of harnessing NaCl saturated natural infusions to produce relatively inexpensive baits for gravid malaria mosquitoes for use in gravid traps. However, at very high concentrations, NaCl will corrode and quickly destroy metallic parts in the traps. More work might be useful to evaluate if smaller amounts of NaCl can still improve the odour plume and reduce damage to the traps.

The high efficiency of BG-Sentinel gravid mosquito traps baited with NaCl saturated soil infusions in collecting gravid *An. gambiae s.s*. suggests their potential use in the field as an odour-baited gravid trap. The trap does not damage specimens, making it ideal for sampling wild mosquito populations in studies that require intact specimens or requires mosquitoes to be captured alive. The trap has been the subject of many explorative evaluations with host-seeking mosquitoes [54, 55] proving its versatility and effectiveness. This study now shows that with only little modifications it has potential for collecting gravid mosquitoes too. Its potential use in large-scale ecological studies or in vector control programmes should to be evaluated in natural field conditions.

## Conclusion

In summary this study (1) describes an efficient bioassay tool and potential new odour-baited trap for gravid females of the *An. gambiae* species complex; (2) provides evidence for the strong involvement of olfaction in the location and selection of potential breeding sites by *An. gambiae s.s.*; and (3) describes the compulsive response of gravid females to attractive chemical cues. Research now needs to be invested in analysing the volatile chemical headspace of the attractive soil infusion to identify attractant semiochemicals for oviposition in *An. gambiae* s.s..
